# Response to Climate Change: Evaluation of Methane Emissions in Northern Australian Beef Cattle on a High Quality Diet Supplemented with *Desmanthus* Using Open-Circuit Respiration Chambers and GreenFeed Emission Monitoring Systems

**DOI:** 10.3390/biology10090943

**Published:** 2021-09-21

**Authors:** Bénédicte Suybeng, Felista W. Mwangi, Christopher S. McSweeney, Edward Charmley, Christopher P. Gardiner, Bunmi S. Malau-Aduli, Aduli E. O. Malau-Aduli

**Affiliations:** 1 Animal Genetics and Nutrition, Veterinary Sciences Discipline, College of Public Health, Medical and Veterinary Sciences, Division of Tropical Health and Medicine, James Cook University, Townsville, QLD 4811, Australia; benedicte.suybeng@my.jcu.edu.au (B.S.); felista.mwangi@my.jcu.edu.au (F.W.M.); christopher.gardiner@jcu.edu.au (C.P.G.); 2 CSIRO, Agriculture and Food, Queensland Bioscience Precinct, St. Lucia, QLD 4067, Australia; chris.mcsweeney@csiro.au; 3 CSIRO, Agriculture and Food, Private Mail Bag Aitkenvale, Australian Tropical Sciences and Innovation Precinct, James Cook University, Townsville, QLD 4811, Australia; ed.charmley@csiro.au; 4 College of Medicine and Dentistry, Division of Tropical Health and Medicine, James Cook University, Townsville, QLD 4811, Australia; bunmi.malauaduli@jcu.edu.au

**Keywords:** methane emission, mitigation, climate change, tannins, *Desmanthus bicornutus*, *Desmanthus leptophyllus*, *Desmanthus virgatus*, polyethylene glycol, GreenFeed, tropical beef cattle

## Abstract

**Simple Summary:**

The beef industry in Northern Australia is characterized by an extensive grazing system in dry tropical rangelands defined by climate change indices of very low rainfall, a prolonged dry season and feeds of low nutritive value. In response, beef cattle need to be more efficient in converting the available drought-tolerant feeds to muscle, in an attempt to minimize greenhouse gas emissions. This study addressed the problem of reducing methane emissions from tropical beef cattle with the goal of decreasing the impact of climate change and greenhouse gas emissions in Northern Australia. The primary objective was to compare the effect of supplementing tropical beef cattle with both good quality lucerne and poor quality hay with increasing levels of different *Desmanthus* cultivars on in vivo methane emission. The results showed that in tropical beef cattle on high-quality diets, irrespective of cultivar and emission evaluation method, *Desmanthus* does not reduce methane emissions.

**Abstract:**

The main objective of this study was to compare the effect of supplementing beef cattle with *Desmanthus virgatus* cv. JCU2, *D. bicornutus* cv. JCU4, *D. leptophyllus* cv. JCU7 and lucerne on in vivo methane (CH_4_) emissions measured by open-circuit respiration chambers (OC) or the GreenFeed emission monitoring (GEM) system. Experiment 1 employed OC and utilized sixteen yearling Brangus steers fed a basal diet of *Rhodes grass* (*Chloris gayana*) hay in four treatments—the three *Desmanthus* cultivars and lucerne (*Medicago sativa*) at 30% dry matter intake (DMI). Polyethylene glycol (PEG) was added to the diets to neutralize tannin binding and explore the effect on CH_4_ emissions. Experiment 2 employed GEM and utilized forty-eight animals allocated to four treatments including a basal diet of *Rhodes grass* hay plus the three *Desmanthus* cultivars in equal proportions at 0%, 15%, 30% and 45% DMI. Lucerne was added to equilibrate crude protein content in all treatments. Experiment 1 showed no difference in CH_4_ emissions between the *Desmanthus* cultivars, between *Desmanthus* and lucerne or between *Desmanthus* and the basal diet. Experiment 2 showed an increase in CH_4_ emissions in the three levels containing *Desmanthus*. It is concluded that on high-quality diets, *Desmanthus* does not reduce CH_4_ emissions.

## 1. Introduction

The global greenhouse gas emissions from livestock supply chains represent 14.5 percent of all human-induced emissions. Consequently, the livestock sector plays an important part in climate change [1]. Enteric methane (CH_4_) produced in the gastrointestinal tract of livestock is the single largest source of anthropogenic CH_4_ [2]. In tropical and subtropical environments such as northern Australia, the poorly digestible pastures with high C:N ratios induce low livestock productivity and increase rumen CH_4_ emissions [3,4]. Previous in vitro [5,6] and in vivo [7] studies showed a decrease in CH_4_ emissions due to dietary supplementation with *Desmanthus*, a tropical forage legume. In vitro studies with *Desmanthus* reported cultivar-dependent differences in CH_4_ emissions. Durmic et al. [6] reported lower CH_4_ emissions compared to the mean emissions from 23 tropical grasses of 48%, 41% and 45% for cultivar JCU1 (*Desmanthus leptophyllus*), cv. JCU2 (*D. virgatus*) and cv. JCU4 (*D. bicornutus*), respectively. Vandermeulen et al. [5] found significantly higher CH_4_ emissions with cv. JCU1 (+33%) and cv. JCU2 (+5%) compared to cv. JCU4. However with in vivo studies, Suybeng et al. [7] did not find any significant difference in CH_4_ yield between JCU1 and JCU4. Vandermeulen et al. [5] attributed the in vitro reduction in CH_4_ emissions to the presence of secondary compounds such as hydrolysable tannins (HT), condensed tannins (CT) and/or their combinations in *Desmanthus spp*. Secondary plant compounds such as phenols, which include CT and HT, have an important role in feeding strategies to mitigate CH_4_ emissions from ruminants [8,9]. Suybeng et al. [7] reported a positive influence of the tannins in *Desmanthus* that bind to proteins in the rumen, increase N utilization and reduce CH_4_ emissions. However, the antimethanogenic property of the tannins in *Desmanthus* was inconclusive as the addition of tannin binder polyethylene glycol (PEG) did not affect CH_4_ emissions [7]. The observed reduction in CH_4_ emissions was attributed to the positive effect of *Desmanthus* on rumen fermentation as the feed quality increased with an increasing level of *Desmanthus* in the diet. It has been reported that a lower quality diet increases CH_4_ yield [10,11]. Gaviria-Uribe et al. [10] reported an inverse relationship between CH_4_ yield and DM and OM digestibility. Therefore, a comparative in vivo study utilizing a non-tannin treatment and another treatment including *Desmanthus* with a similar nutritive value, would clarify the impact of *Desmanthus* and secondary plant compounds on CH_4_ emissions. Furthermore, due to discrepancies in CH_4_ emissions between previous in vitro and in vivo results comparing several *Desmanthus* cultivars, an in vivo pen feeding trial with a larger group of animals to test the cultivar effect on CH_4_ emissions would also provide further clarity. Lucerne (*Medicago sativa*) is a commonly used temperate perennial legume in southern Queensland and northern New South Wales for its high quality crude protein [12,13]. The comparative impact of supplementing beef cattle on a basal diet of *Rhodes grass* with varying levels of the tropical forage legume *Desmanthus* spp. and the temperate legume lucerne on CH_4_ emissions may fill in this significant knowledge gap in tropical beef cattle nutrition.

Open-circuit respiration chambers (RCs) are considered as the ‘gold standard’ for accurately measuring CH_4_ production from rumen and hindgut fermentation [14,15,16,17]. However, RCs are expensive to construct, technically demanding to operate and maintain, and cannot be used to measure many animals at once [17]. Furthermore, feed intakes are generally lower in RCs with the possibility of inducing higher CH_4_ yields (expressed as g/kg dry matter intake (DMI)) [18,19]. In contrast, the GreenFeed emission monitoring (GEM) system is a patented automated head-chamber system based on spot sampling (2–6 min) of eructated and exhaled gases allowing measurements of enteric CH_4_ production on a large number of animals under on-farm conditions [20]. This technique has minimal labor input and interference with animal behavior and production [21]. Previous studies reported minor differences between RC and GEM methods in average CH_4_ emission values [22]. In this study, the comparative effect of four different dietary inclusion levels of *Desmanthus* on CH_4_ emissions was evaluated using GEM in a pen-based experiment imitating a grazing situation. The overall objectives of this investigation were to compare the antimethanogenic effect of three *Desmanthus* cultivars and the effect of supplementing Brangus steers on a basal diet of *Rhodes grass* with the tropical forage legume *Desmanthus* spp. and the temperate legume lucerne, on CH_4_ emissions. The study tested the hypothesis that increasing the proportion of *Desmanthus* in the diet will reduce CH_4_ emissions when measured by GEM.

## 2. Materials and Methods

### 2.1. Experimental Procedures

Two experiments were conducted at the Commonwealth Industrial and Scientific Research Organization (CSIRO) Lansdown Research Station, Queensland, Australia (19.59° S, 146.84° E) following the Australian Code for the Care and use of Animals for Scientific Purposes (Eighth edition, 2013) and were approved by the CSIRO Queensland Animal Ethics Committee. Experiment 1 took place from the 1 February to 26 June 2020 (Permit Number 2019-32) and aimed to compare the effect of supplementing tropical beef cattle with *Desmanthus* cultivars JCU2, JCU4 and JCU7 or lucerne on in vivo CH_4_ emissions measured by RC. Experiment 2 was carried out from the 17 March to 21 July 2020 (Permit Number 2019-38) and aimed to explore the effect of incrementally supplementing tropical beef cattle with equal proportions of the three *Desmanthus* cultivars on CH_4_ emissions measured by GEM system.

### 2.2. Desmanthus Cultivars

JCU2 (*D. virgatus*) was selected for its rapid growth, seed set, persistence under plant density and grazing relative to other *Desmanthus* cultivars [23,24]. The first flowering days from sowing are around 90.8 days [24]. This cultivar is reported to perform well across a number of environments with buffel grass and native grasses in northern and central Queensland [25]. JCU4 (*D. bicornutus*) was also selected for its persistence and plant density [24]. It is a robust early maturing plant (average of 84 days for the first flowering after sowing) [24,25] that is used for pasture improvement on dark clay soils in semi-arid zones [23]. JCU7 (*D. leptophyllus*) is a late flowering species with limited seed production. It was selected for its leafiness and bulk production [23]. Fresh *Desmanthus* was harvested from a farm located 20 min away from the research station.

### 2.3. Experiment 1

#### 2.3.1. Animals and Treatments

The experimental design of Experiment 1 was described by Suybeng et al. [26]. Briefly, a completely randomized block design was used to allocate sixteen yearling Brangus steers weighing 232 ± 6 kg on average, into four treatments of four animals each based on similar liveweight (LW). The four treatments comprised *Rhodes grass* (*Chloris gayana*) as basal diet plus either lucerne (*Medicago sativa*) hay or one of the 3 species of fresh *Desmanthus* (*D. virgatus* (JCU2), *D. bicornutus* (JCU4) and *D. leptophyllus* (JCU7)) at 30% dry matter (DM). The percentage of lucerne in the diet was adjusted periodically to match and equilibrate the crude protein (CP) content in the diets containing *Desmanthus*. Prior to the start of the trial, 16 out of 20 animals were selected based on temperament. These animals were subjected to a three-week training period during which time they adapted to the respiration chambers. There were five periods in this experiment. The first period constituted the backgrounding period where all the animals were offered *Rhodes grass* for 28 days and adapted to a hay-based diet. The backgrounding period was followed by a 28-day duration where the animals were adapted to either lucerne or one of the *Desmanthus* spp. Thereafter, period length was reduced to 14 days as animals were already adapted to legumes in the diet and the cultivar effects on digestion were considered less than introduction of legumes to a grass diet. It should be noted that as fresh *Desmanthus* was being harvested throughout the study, there was an imperative to keep the trial as short as possible to limit nutritional changes over the growing season. During each of the periods 2, 3 and 4, the animals on *Desmanthus* received each *Desmanthus* cultivar once. A final period was included where all the animals from each group stayed on their same *Desmanthus* cultivar diet from period 4 and 2 animals from each group of 4 animals were supplemented with polyethylene glycol (PEG 4000, Redox Pty Ltd., Minto, NSW, Australia) at 160 g/kg *Desmanthus* DM to annul the bioactivity of tannins. Within each period, animals were fed *ad libitum* (10% uneaten feed after 23 h), then reduced to 90% of *ad libitum* four days before entry into the respiration chambers. The amounts of hay and *Desmanthus* were adjusted daily, weighed out for individual animals and thoroughly mixed immediately before feeding. The animals were fed once daily between 9:30–10:00 a.m.

#### 2.3.2. Measurement of Methane Emissions

Animals were ranked according to weight and divided into four blocks. Within each block, one animal was allocated at random to one treatment. Four respiration chambers were used to measure CH_4_ emissions from individual steers as described by Martinez Fernandez et al. [27] and Suybeng et al. [7]. Four series of measurements were taken in each period over two weeks. In this way, 16 animals were subjected to chamber measurements over 48 h in four groups of four. Start dates for each period were staggered to ensure all animals were on treatments for the same length of time within each period. Within each series, all four treatments were included (one animal per treatment). Briefly, CH_4_ emissions were measured using independent units (23.04 m^3^, 3000 L/min airflow) comprising drinking water and the daily ration in a feed bin. The internal atmosphere of the chambers was maintained at approximately 24 °C, −10 Pa and relative humidity of 50–75%. Methane production was calculated following a correction of the flow rates to measured conditions for temperature and pressure [28]. Methane emissions were monitored continuously by infrared absorption (Servomex 4100, Servomex Group Ltd. Crowborough, UK) for 48 h. Methane production (g CH_4_/day) was determined using the average of two 24 h measurements. DMI in the chamber was reported daily to calculate the CH_4_ emissions according to feed intake (CH_4_ yield expressed as g/kg DMI).

### 2.4. Experiment 2

#### 2.4.1. Animals and Treatments

This experiment was a pen-based feeding trial that ran for 128 days comprising 14 days of adaptation on a basal diet of *Rhodes grass* hay (9% CP) and the remainder on treatment diets comprising the basal diet and varying levels of *Desmanthus* for 114 days of feeding. Forty-eight animals in a completely randomized experimental design with an equal number of cattle in four treatment groups were utilized. Cattle were ranked according to weight and blocked into 12 blocks, with one animal from each block allocated to one of four treatments (0, 15, 30 or 45% *Desmanthus* inclusion in the diet on DM basis) with four animals per pen and three pens per treatment. Three *Desmanthus* cultivars (JCU2, JCU4 and JCU7) were fed in equal proportions at each treatment level. The *Desmanthus* treatments were adjusted with lucerne at 7–14 day intervals to obtain a similar CP content in all four treatments. The treatments were mixed thoroughly daily before feeding. Four cattle from the same treatment were allocated to a pen. Each pen space was 60 m^2^ and equipped with a feed bunk, shade and water.

#### 2.4.2. Measurement of Methane Emissions

Methane emissions were measured with 4 GEM systems (GEM, C-Lock Inc., Rapid City, SD, USA). Each GEM unit was allocated to the sequential measurement of three pens for 28 days followed by three periods of 10 days. In each period, one unit was available for 4 animals per pen. Thus, the animals in each pen were monitored by the same GEM unit on two occasions (27 and 10 days’ duration). The GEM units were solar powered and their operation was initiated when the animal placed its head inside the hood. A radio frequency identification (RFID) reader identified the animal’s ear tag which started the measurement. During visits, enteric gas emissions were measured and pelletized bait feed (Barastoc Calm Performer, Ridley Agriproducts, Harristown, QLD, Australia) was dropped in each session. With the CP concentration of the pellets being around 11%, no pellet effect on the treatments was expected. The details of GEM design, operation and analyses has been described by Hammond et al. [29]. Briefly, an animal puts its head and shoulders into a semi-enclosed space to access feed pellets. Air is drawn past the animal and subsampled for analysis to determine CH_4_ concentrations and CO_2_ after correction for background concentrations. Only animal visits of more than 2 min were kept for analysis. The GEM was programmed using C-Lock Inc, Software to deliver a maximum of 4 rotations of a feed dispensing cup delivering approximately 50 g of pellet (as fed) per rotation, with intervals of 45 s between each rotation so that 200 g of pellet was delivered during each visit. A maximum of 5 visits per day (24 h) was allowed with a minimum of 4.8 h required in between visits. The number of drops per animal was recorded and added to the DMI. In total, 2844 GreenFeed visits (an average of 33 visits/animal and 2.8 visits/day/animal) were collected and processed from this experiment. An average of 36, 26, 35 and 36 visits for the 0%, 15%, 30% and 45% *Desmanthus* levels respectively, was recorded.

### 2.5. Feed Chemical Composition

The same forages were used in both trials. A scanning monochromator (model 6500, NIRSystem, Inc., Silver Spring, MD, USA) was used to determine the chemical composition of the feed samples by near-infrared reflectance spectroscopy (NIRS) and calibration equations developed by CSIRO Agriculture [30] using ISI Software (Infrasoft International, Port Matilda, PA, USA) as described by Durmic et al. [6]. Nutritive value of diets was presented in a previous paper [26] and are reported in Table 1.

Metabolizable energy (ME) was calculated as DMD × 0.172–1.707 [31] from the NIRS data. The CP, acid detergent fiber (ADF), neutral detergent fiber (NDF) and ME intake were calculated as the CP, ADF, NDF, ME of the dry feed offered minus the CP, ADF, NDF, ME of the dry feed refused after 24 h for Experiment 1 and after one week for Experiment 2.

### 2.6. Plant Extraction Procedure and Analysis of Secondary Compounds

Fresh samples of the three *Desmanthus* cultivars, *Rhodes grass* and lucerne were sampled every week, stored at −20 °C, freeze-dried at −50 °C for 3 days in a freeze dryer (Epsilon 2-6D LSCplus, Christ, Osterode am Harz, Göttingen, Germany) and ground to pass a 1 mm screen using an Ultra Centrifugal Mill ZM 200 (Retsch GmbH, Haan, Germany) and kept at room temperature (20 °C) [32]. A 0.25 mm sieve was used to pass the freeze-dried material before analysis. The laboratory procedure of Terrill et al. [32] was followed for tannin extraction, except that the supernatant was increased to 300 µL total volume in distilled water.

An estimation of the proanthocyanidin concentration (CT) was determined by the Butanol-HCl-Fe^III^ method using purified *Desmanthus* CT as the standard with absorbance detected at 550 nm [33,34]. Condensed tannin (CT) was purified on Sephadex LH-20 as defined by Wolfe et al. [35]. The Folin−Ciocalteu method was used to determine the total phenolic (TP) concentration with catechin as the standard [33].

### 2.7. Dry Matter Intake and Liveweight

In Experiment 1, the LW of each animal was recorded weekly and individual DMI calculated by the difference between offered and residual feed after 24 h. In Experiment 2, the LW of each animal was recorded fortnightly and the DMI per pen was calculated by the difference between offered and residual feed after 24 h. These values were used to calculate the DMI expressed as % LW. CH_4_ yield was computed on per kg DMI basis and CH_4_ emissions per kg LW.

### 2.8. Statistical Analyses

R (Rstudio version 1.3.1056, R Core Team (2013). R: A language and environment for statistical computing. R Foundation for Statistical Computing, Vienna, Austria, ISBN 3-900051-07-0, URL http://www.R-project.org/ (accessed on 19 September 2021) was used to analyse all data with the ‘dplyr’ [36], ‘nlme’ [37], ‘lme4’ [38], ‘car’ [39] and ‘multcomp’ [40] packages. Effects were considered significant at *p* < 0.05.

In Experiment 1, a linear mixed model procedure was applied to compare the intakes, phenolic concentrations and CH_4_ emissions between the three *Desmanthus* spp. (JCU2, JCU4 and JCU7), between lucerne and the three *Desmanthus* spp. and between the backgrounding diet and three *Desmanthus* spp. The intakes, phenolic concentrations and CH_4_ emissions were the dependent variables, whilst the treatments were the fixed effects and individual animals nested within blocks were the random effects.

Y_ijkl_ = µ + A_i(l)_ + T_j_ + P_k_ + B_l_ + ξ_ijkl_

where Y_ijkl_ is the response variable of the i^th^ animal (i = 1 to 16) nested in the l^th^ block (l = 1 to 4) that received the j^th^ treatment (j = baseline, lucerne, JCU2, JCU4 and JCU7) during the k^th^ period (k = 1 to 4), µ is the overall mean of all observations, A_i(l)_ is the random effect of the experimental animal nested in the l^th^ block, T_j_ is the fixed effect of the treatment, P_k_ is the fixed effect of the period, B_l_ is the fixed effect of the block, and ξ_ijkl_ is the random error component.

The same model was also used to examine the impact of supplementing with PEG on these variables except that only the data from the animals of the *Desmanthus* diet in period 5 were analyzed and the fixed effect was the presence or absence of PEG. The model was fitted using the restricted maximum likelihood (REML) procedure.

In Experiment 2, a linear mixed model procedure was utilized to compare the intakes and CH_4_ emissions between the 4 treatments. The intakes and CH_4_ emissions were the dependent variables, whilst the four treatments were the fixed effects and individual animals nested within pens were the random effects.

Y_ijkl_ = µ + A_i(l)_ + T_j_ + P_k_ + Q_l_ + ξ_ijkl_

where Y_ijkl_ is the response variable of the i^th^ animal (i = 1 to 48) nested in the l^th^ pen (l = 1 to 12) that received the j^th^ treatment (j = 0%, 15%, 30%, 45% *Desmanthus*) during the k^th^ period (k = 1, 2), µ is the overall mean of all observations, A_i(l)_ is the random effect of the experimental animal nested in the l^th^ pen, T_j_ is the fixed effect of the treatment, P_k_ is the fixed effect of the period, Q_l_ is the fixed effect of the pen, and ξ_ijkl_ is the random error component.

When significant differences were detected, mean separation by pairwise comparison was carried out using the Tukey test.

## 3. Results

### 3.1. Experiment 1

#### 3.1.1. Chemical Composition of the Diets

**Figure 1:**
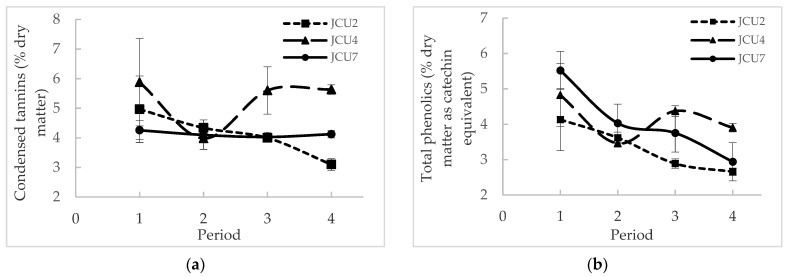
Variation in (**a**) condensed tannins (% dry matter) and (**b**) total phenolics (% dry matter as catechin equivalent) throughout the feeding period.

The nutrient and secondary compound intakes in the diet are given in Table 1. The intakes of the three *Desmanthus* cultivars, CT and TP in the diet were similar. Although the CT in the diet was similar in the three *Desmanthus* diets, Figure 1 shows that the CT concentration in JCU4 (5.27 ± 0.43% DM) was significantly higher than in JCU2 (4.10 ± 0.309% DM) and JCU7 (4.13 ± 0.11% DM) (*p* = 0.014). However, the TP concentration in the three *Desmanthus* cultivars was not significantly different (3.32 ± 0.31%, 4.14 ± 0.32% and 4.06 ± 0.31% DM as catechin equivalent for JCU2, JCU4 and JCU7, respectively). The TP in JCU2, JCU4 and JCU7 and the CT in JCU2 and JCU4 were not significantly different throughout the trial (Figure 1). The CT in JCU7 was significantly higher in period 1 compared to period 4. The CT and TP in the diet were significantly lower in the backgrounding and lucerne diets compared to the *Desmanthus* diets (Table 2).

The intakes of DM, CP and ME were significantly lower in the backgrounding diet than in the *Desmanthus* diets (Table 2). The animals on the lucerne diet had a higher DMI, CP, ADF, NDF and ME intakes than the animals on the three *Desmanthus* diets. Although the DMI was higher for the animals on the lucerne diet, the DMI/kg LW was similar for all diets.

#### 3.1.2. Impact of Diet on Methane Emissions

The CH_4_ production, yield and CH_4_ expressed as g/kg LW were not significantly different between the three *Desmanthus* treatments (Table 3). The CH_4_ production (g/d) was lower in the backgrounding diet and higher in the animals fed lucerne compared to the *Desmanthus* treatments. However, there was no significant difference in the CH_4_ yield (g/kg DMI) between all the treatments. The CH_4_ expressed as g/kg LW was not significantly different between the backgrounding and the *Desmanthus* diets. However, CH_4_ expressed as g/kg LW was significantly higher in the animals fed lucerne compared to the animals on the *Desmanthus* diets.

**Figure 2:**
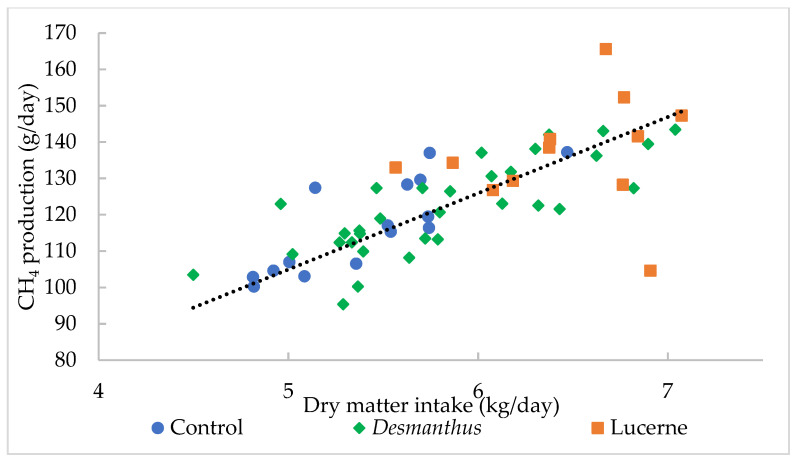
The relationship between dry matter intake (kg /day) and CH_4_ production (g/day). The relationship can be described as CH_4_ production (g/day) = 20.99X, where X = dry matter intake (kg/day) R^2^ = 0.73, *p* < 0.001.

Methane production was highly correlated to DMI (R^2^ = 0.73) (Figure 2). One kg increase in DMI per day increased CH_4_ production by 20.99 g/day. There was no correlation between CH_4_ yield and CT diet (*p* = 0.53), TP diet (*p* = 0.39), percentage of *Desmanthus* in the diet (*p* = 0.96) or *Desmanthus* DMI (*p* = 0.97).

#### 3.1.3. Effect of Polyethylene Glycol on Nutritive Intakes, Phenolics, Tannins Concentrations and Methane Emissions

The addition of PEG to the diet significantly increased the CH_4_ production expressed as g/day (Table 4). The PEG addition did not have any effect on the intakes and CH_4_ yield (g/kg DMI), expressed as g/kg LW.

### 3.2. Experiment 2

**Figure 3:**
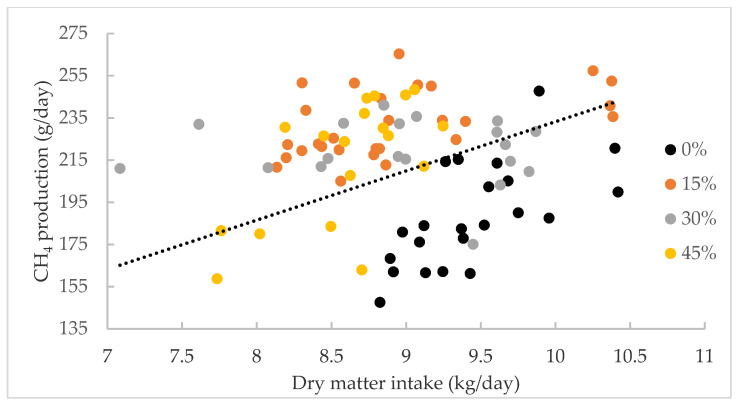
The relationship between dry matter intake (kg/day) and CH_4_ production (g/day). The relationship can be described as CH_4_ production (g/day) = 23.32X, where X = dry matter intake (kg/day) R^2^ = 0.68, *p* = 0.028.

The effect of the level of inclusion of *Desmanthus* cultivars on intake, CH_4_ production and yield are presented in Table 5. There was no significant difference in the intake of DM (expressed as kg/day or g/kg LW) or CP between the four diets. The ADF intake was significantly lower in the diet containing 45% *Desmanthus* compared to the 0% *Desmanthus* diet. NDF intake was also significantly lower in the 30 and 45% *Desmanthus* diets compared to the 0% *Desmanthus* treatment. ME intake significantly decreased with the increasing level of *Desmanthus* in the diet. Methane production was significantly higher for the 15% and 30% *Desmanthus* inclusion rates compared to the 0% inclusion rate. However, CH_4_ production for the 45% inclusion rate was not different to other inclusion levels. The CH_4_ yield followed a similar pattern, except that CH_4_ yield for the 30% *Desmanthus* inclusion rate was lower than that for the 15% inclusion level. Methane production was correlated to DMI (Figure 3). One kg of DMI increase induced an increase in CH_4_ production of 23.32 g/day.

## 4. Discussion

### 4.1. Experiment 1

#### 4.1.1. Comparison of Methane Emissions between the Desmanthus Cultivars

In general, the chemical composition of *Desmanthus* in the current study suggested lower nutritive value, as evidenced by lower CP and higher NDF compared to the previous in vitro studies conducted by Vandermeulen et al. [5]. For instance, they found a CP concentration of 12.6% DM and 13.8% DM for JCU2 and JCU4, respectively, and an NDF concentration of 52.5% DM and 47.8% DM for JCU2 and JCU4, respectively, after 51 days regrowth. In JCU4, Suybeng et al. [7] reported CP and NDF concentrations of 14.6 and 58.3% DM, respectively, after six weeks regrowth. The *Desmanthus* in our present study had a CP concentration of 10.3% DM and 13.0% DM for JCU2 and JCU4, respectively, and a NDF concentration of 57.5% DM and 53.1% DM for JCU2 and JCU4, respectively, after four to six weeks regrowth [26]. These data collectively demonstrate that the nutritive value of *Desmanthus* can vary widely depending on the cultivar/species, stage of growth, edaphic and climatic conditions. However, JCU2 appears to be of a lower quality than JCU4 [5,6].

The current study showed no significant difference in nutrient intake between the *Desmanthus* diets, presumably due to similar nutritive values. The concentration of TP and CT in the plants in this experiment were higher than the values reported by Suybeng et al. [7], where the same standards were used, although the absorbance of the same samples used for the standard curve differed between the two studies, being higher in the current trial. This observation highlights the fact that the TP and CT concentrations can only be compared within a short period of time in the same laboratory [35]. However, the higher CT concentration in JCU4 corroborates the report of Vandermeulen et al. [5] where they found a higher CT concentration in JCU4 during winter compared to JCU1 and JCU2 (Table 6). They also reported a significantly lower concentration of TP and CT in JCU2 compared to JCU1 and JCU4 in contrast to our current findings. Gonzalez-V et al. [41] showed an increase in tannin concentration with maturity (from 60 days to 120 days after planting) and a higher tannin concentration in the leaves compared to the stems. The decrease in CT in JCU7 from periods 1 to 4 may be due to a decrease in the leaf to stem ratio or the harvesting of a younger regrowth towards the end of the trial. McMahon et al. [42] explained the difference in the concentration of CT as a function of plant maturity due to the activities of four enzymes in the CT biosynthetic pathway which are maximal in young, unexpended leaves of sainfoin and decline or are absent in older leaves. However, the tannin concentration in the plant is also influenced by environmental conditions. For instance, Top et al. [43] found that the green leaf of *Quercus rubica* produced 50% more tannins when grown in dry conditions compared to wet conditions.

There was no significant difference in CH_4_ emissions between the *Desmanthus* cultivars which corroborates the in vivo findings of Suybeng et al. [7], but contradict the in vitro results of Vandermeulen et al. [5] that showed higher CH_4_ emissions expressed as mL/g OM fermented with JCU2 compared to JCU1 and JCU4 after 72 h (Table 6). On the other hand, Durmic et al. [6] showed similar in vitro CH_4_ emissions expressed as mL/g DMI with JCU2 and JCU4 (29.2 and 29.7 mL/g DMI for JCU2 and JCU4, respectively), and lower CH_4_ production with JCU1 (24.2 mL/g DMI).

Data from a limited number of studies with these *Desmanthus* cultivars demonstrate the inherent variability in nutritive value, phenolic compounds and methane production both in vitro and in vivo. While there is evidence to support a relationship between phenolic compound concentration and methane production [5,7], it is by no means a clear relationship [9]. In vivo studies require a long feeding period, during which chemical composition may change [44].

#### 4.1.2. Comparison of Methane Emissions between the Backgrounding and *Desmanthus* Diets

The lower DMI during the backgrounding period was attributed to the experimental design of the feeding trial as the backgrounding period occurred at the start of the experiment when the animals were smaller. However, the DMI expressed as g/kg LW was not significantly different between the backgrounding and *Desmanthus* diets. The lower DMI expressed as kg/day induced a lower CH_4_ production compared to the *Desmanthus* diets as it is correlated to DMI. The current study showed a linear increase in CH_4_ production with an increase in DMI with the slope of the equation being 20.99, which corroborates previous findings by Charmley et al. [45] who reported a slope of 20.7. Benaouda et al. [46] stated that DMI can explain 78% of the variation in CH_4_ emissions and account for up to 92% of the variation when only RC data were used [45]. Boadi and Wittenberg [11] reported a strong correlation (r = 0.8) between DMI and CH_4_ production with DMI accounting for 64% of the daily variation in CH_4_ production. Feed intake is therefore one of the key factors accurately accounting for variation in CH_4_ emissions in cattle [46,47].

Suybeng et al. [7] reported an 8% decrease in CH_4_ yield (from 19.1 to 17.5 g/kg DMI) from 0% to 31% *Desmanthus* inclusion in the diet in contrast to this current study where no significant difference was observed between the backgrounding and the *Desmanthus* diets. The presence of tannins in *Desmanthus* is frequently cited as the cause for reduction in methane emissions and was a possible contributory factor, but this will be discussed in more detail in subsequent sections. The lack of difference in CH_4_ yield between the backgrounding and *Desmanthus* diets could be due to similar NDF intakes in the backgrounding and *Desmanthus* diets since NDF intake is directly related with CH_4_ emissions [10].

Our results contradict the in vitro study findings by Vandermeulen et al. [5] who reported a decrease in CH_4_ emissions with the JCU2 and JCU4 *Desmanthus* cultivars compared to *Rhodes grass*. The difference in CH_4_ emissions in their study can be attributed to the higher dietary NDF in *Rhodes grass* compared to the *Desmanthus* cultivars. In the current study, even though the *Desmanthus* diets were of a higher quality than the backgrounding treatment (higher CP intake) and contained secondary plant compounds (two aspects that would be expected to reduce methane emissions), there was still no difference in CH_4_ emissions between the treatments.

#### 4.1.3. Comparison of the Methane Emissions between the Lucerne and *Desmanthus* Diets

There is ample evidence to show that improving the nutritive value of diets reduces methane production (if intake remains unaltered) and methane yield and this holds true for temperate and tropical diets and forage-based and concentrate-based diets. There is also evidence to suggest that bioactive compounds found in tropical legumes, such as *Desmanthus*, can reduce methane production and yield. In the current study, these two drivers of methane production are working in opposing directions. That is, the lower nutritive value of *Desmanthus* versus lucerne may be serving to increase methane production from *Desmanthus* while the bioactive may be serving to reduce methane production from *Desmanthus*. Thus, the overall effect is that there was no difference in methane production or yield between the lucerne and *Desmanthus* treatments.

The lack of significant difference in CH_4_ emissions between the lucerne and *Desmanthus* diets in the current in vivo study contradicts previous in vitro study findings by Durmic et al. [6] that reported a 27% decrease in CH_4_ emissions from *Desmanthus* compared to lucerne. In that study, the lucerne quality was similar to the *Desmanthus*, whereas in the current study the *Desmanthus* cultivars were of lower quality than the lucerne. This might explain the difference between the current study and the study conducted by Durmic et al. [6]; the diet quality effect in the current study is negating any possible bioactive effect.

The increase in DMI observed in the lucerne diet can be attributed to the higher digestibility of lucerne (65.2%) compared to *Desmanthus* spp. (49.6%) [26]. Kennedy and Charmley [48] reported an increase in both dry organic matter intake (DOMI) and CH_4_ production in steers fed lucerne compared to cattle on tropical grasses such as speargrass (*Heteropogon contortus*), buffel grass (*Cenchrus ciliaris*), bisset grass (*Bothriochloa insculpta*), Mitchell grass (*Astrebla lappacea, Astrebla elymoides*), *Rhodes grass* (*Chloris gayana*) or tropical legumes such as Burgundy bean (*Macroptilium bracteatum*) or Stylo (*Stylosanthes hamata*). They found a decrease in methane emissions expressed as g per kg DOMI by up to 26% and 10% in animals given high quality lucerne compared to the animals given poorer quality buffel grass and Stylo, respectively. The increase in DOMI can be explained by the higher digestibility of lucerne compared to tropical grasses and legumes which induced a rise in digestive efficiency as reflected by a lesser loss of energy to CH_4_ production. Gaviria-Uribe et al. [10] also showed that methane emissions expressed per kg of DMI and methane intensity expressed per unit liveweight gain were significantly higher in low quality feed composed of Cayman (*Urochloa hybrid*) compared to Cayman mixed with *Leucaena leucocephala* or *Leucaena diversifolia*. Low concentrations of NDF and ADF are characteristic of high-quality forages. Digestible NDF proportions of about 15 and 25 percentage units are optimal for legumes and grasses, respectively, hence, high quality forages are digested quickly, a process which minimizes rumen/gut fill and permits maximum dry matter intake [49,50,51,52].

The lower DMI in the *Desmanthus* diets can also be due to lesser palatability compared to lucerne. Palatability is defined as the characteristic of a feed indicating its acceptability regarding gustatory, olfactory or visual senses [44]. It affects an animal’s preference for a given feed when offered choice and the rate of eating and intake when offered a single feed [49]. Palatability is often based on astringency associated with CT-protein complexes formed from proteins in saliva. Therefore, the greater the proteins bound by CT, the greater the astringency and the lower the palatability [53]. Usually, a depressed intake is seen at dietary CT concentrations exceeding 5% of DM. However, it is possible that intake may be depressed at concentrations less than 5% of DM when the CTs are more effective at protein binding and at concentrations greater than 5% DM when the CTs are less effective [53]. In the in vivo study by Suybeng et al. [7] with Droughtmaster steers supplemented with 31% *Desmanthus* on DM basis, there was no decrease in DMI.

The lack of reduction in CH_4_ yield in the *Desmanthus* diet can also be explained by the low concentration of tannins (lower than 2% DM for both CT and TP). Previous studies with low or moderate tannin concentrations in the diet failed to reduce enteric CH_4_ emissions in cattle. For instance, Beauchemin et al. [54] observed a protein-binding effect, but reported no reduction in CH_4_ emissions in growing cattle supplemented with 2% DM quebracho tannin extract. Moreover, a recent in vitro study conducted by Thirumeignanam et al. [55] reported a significant decrease in CH_4_ production expressed as ml/g/h on a hedge lucerne (*D. virgatus*) silage diet supplemented with 3 and 4% (*w*/*w*) tannin as tannic acid equivalent from *Acacia nilotica* pods with goat rumen fluid compared to the diet containing 1, 2 and 5% (*w*/*w*) tannin as tannic acid.

Therefore, the higher quality and digestibility of lucerne induced a higher nutrient intake compared to the *Desmanthus* treatments. The higher quality of the lucerne diet may have reduced the methane yields relative to the lower quality diets that contained *Desmanthus*. This effect could potentially mask a tannin effect on reducing methane emissions from *Desmanthus* cultivars. Thus, two different processes, both acting on methane emissions, may have counteracted one another. The possibility that the secondary plant compounds in the *Desmanthus* were affecting CH_4_ emissions cannot be completely ruled out.

#### 4.1.4. Effect of Polyethylene Glycol on Methane Emissions

Methane production was significantly higher with PEG addition, but no difference in CH_4_ yield was observed. This observation corroborates the study conducted by Suybeng et al. [7], where they reported no difference in CH_4_ yield in the presence of PEG in a diet containing 22% *Desmanthus* DM. However, the results contradict some previous studies that showed an increase in CH_4_ yields expressed as L per kg DMI and mM per g of DM with the addition of PEG in a diet containing tannins at 15% DM [56] and 25% DM [57], respectively. Moreover, Fagundes et al. [3] did not find any correlation between CT concentration and biological effect; the biological effect of tannins being an increase in gas production when a binding agent is added [58]. They indicated that chemical analysis alone would not predict CT bioactivity which can be related to structure as much as concentration of the molecule. Nevertheless, they found a link between TP content and biological effect. For instance, the species with the greatest biological effect had the highest phenolic content. In the current study, neither CT nor TP was correlated with CH_4_ emissions. However, when their biological activity was eliminated with PEG addition, a small elevated CH_4_ yield response was observed in the presence of *Desmanthus*. The limited evidence for a tannin effect suggests tannins may have been inhibiting methanogenesis, but the evidence is not strong, possibly due to the low levels of tannins in these *Desmanthus* cultivars. It is important to note that other secondary plant metabolites can contribute to reducing the methane emissions from ruminants, such as saponins, essential oils and flavonoids [59], which might be present in *Desmanthus*.

### 4.2. Experiment 2—Effect of Level of Inclusion of Desmanthus Cultivars on Intake, Methane Production and Yield

The results of experiment 2 showed no significant difference in DM and CP intakes in comparison to Experiment 1 where both intakes were higher in the lucerne diet. These observations are contrary to the report of Suybeng et al. [7] showing a linear increase in DMI with increasing level of *Desmanthus* inclusion in the diet. In contrast, the ADF and NDF intakes decreased with a trend towards lower DMI as *Desmanthus* proportion in the diet increased. Metabolizable energy decreased with the increasing level of *Desmanthus* as ME was lower in the *Desmanthus* spp. compared to lucerne. Similarly, the significantly higher CH_4_ yields in the 15 and 30% *Desmanthus* treatments compared to the 0% *Desmanthus* (backgrounding) does not align with previous studies either showing a decrease or comparable CH_4_ emissions with and without *Desmanthus* [5,6,7]. The difference in CH_4_ emissions compared to previous studies might be due to limitations associated with the GreenFeed units. Arbre et al. [60] stated that to obtain a correlation of r = 0.70 for CH_4_ yield (g/kg DMI), a 17-day period for GreenFeed monitoring was necessary, along with a number of animals of 6–8 per group to be able to detect a difference of 20% in CH_4_ yield between the treatments. Manafiazar et al. [61] also reported that 7 to 14 days with a minimum of 20 samples per animal were necessary to produce repeatable and reliable averaged CH_4_ and CO_2_ emissions correlated with DMI. In the present study, even though the first period lasted 28 days, the number of animals per treatment in each period was four. Furthermore, if only the over 20 visits per animal were used, CH_4_ yield would have stayed similar (20.2, 26.4, 24.1 and 24.7 for the 0, 15, 30 and 45% *Desmanthus* diets, respectively). Previous studies suggested a lower repeatability when averaged over a long period compared to a shorter monitoring period due to changes in the animals’ physiological status, which can induce between-period variability [62]. For instance, Denninger et al. [63] showed an increase in repeatability of up to 0.68 when the measurement period was extended from 7 to 14 days, but showed a decrease in repeatability when the measurement period was further extended to 28 days. Arthur et al. [64] reported significantly less heterogeneous variances by taking the records with a minimum of 3 min GEM visit duration instead of 2 min. In the current study, by taking only the measurements with a minimum of 3 min GEM duration, CH_4_ yield was significantly lower in the 0% *Desmanthus* treatment (20.1 g/kg DMI) compared to the three *Desmanthus* treatments (26.8, 25.2 and 24.9 g/kg DMI for the 15, 30 and 45%, respectively). Hristov and Melgar [65] also reported the need for sufficient number of observations covering the entire 24 h feeding cycle to have representative emission estimates using the GEM system, because measurements using GEM depended on the time of measurement relative to the time of feeding. The increase in CH_4_ emissions with an increase in *Desmanthus* level could also be explained by the experimental design, as most of the animals in the backgrounding (0% *Desmanthus* DM) treatment were measured by the same unit compared to the other treatments that were measured by two different units. Consequently, comparisons between the GEM units is not feasible.

### 4.3. Comparison between Open-Circuit and GreenFeed Emission Monitoring Systems

The CH_4_ yield measured with the GEM was quantitatively higher compared to RC. However, the slope of the response in methane to DMI (0.0233) was quite similar to Experiment 1 (0.0210). Previous studies showed mixed results regarding the comparison between RC and GEM unit measurements. For instance, Alemu et al. [66] reported a significantly higher CH_4_ yield measured by the GEM system (28.5 g/kg DMI) compared to the RC results (26.5 g/kg DMI) for the same animals. They explained this difference by the decrease in DMI in the RC that can vary between 10 to 19% due to the stress associated with change of environment and the decreased energy expenditure in the respiration chamber [19,67]. Huhtanen et al. [20] also reported greater CH_4_ production measured by GEM (13 g/day) than those measured by RC. However, Doreau et al. [22] reported a lower CH_4_ emission for GEM than the RC by 14% on average. They attributed this difference to flatus and feces that are measured in the RC and not by the GEM. Although only 2–4% of enteric production is caused by flatulence, they explained the underestimation of CH_4_ emissions with GEM due to missing the postprandial peak of emission as the proportion of visits to the GEM is low during main meals [68]. The correlation between GEM and RC measurements are inconsistent, with values fluctuating between 0.37 and 0.32 for dry cows [22], 0.60 and 0.85 for cattle [69] and between 0.10 and 0.058 for heifers [29]. However, Huhtanen et al. [20] showed a good relationship in CH_4_ production measured by RC and GEM (R^2^ = 0.92) when they studied 20 direct comparisons. Previous studies generally agreed that there were minor differences between the two methods for average values, but individual correlations may limit their interchangeability for determining the gas emissions of individual animals [22].

## 5. Conclusions

*Desmanthus virgatus* (JCU2), *D. bicornutus* (JCU4) and *D. leptophyllus* (JCU7) showed no difference in secondary plant compound concentrations (CT and TP) or in CH_4_ emissions. Despite the presence of these compounds in *Desmanthus* spp., no difference was observed in CH_4_ yield between the *Desmanthus* treatments, the backgrounding (*Rhodes grass*) or lucerne diets when CH_4_ was measured with RC. The similar CH_4_ emissions between the lucerne and *Desmanthus* diets may be attributed to the higher quality and digestibility of lucerne compared to *Desmanthus* and to the low level of secondary plant compounds in the diet. The absence of tannin effect on CH_4_ emissions was highlighted with the addition of PEG which did not show any difference.

An increase in CH_4_ yield with a *Desmanthus* inclusion level of 15, 30 and 45% DM in the diet was observed when CH_4_ was measured with GEM, compared to the *Rhodes grass* and lucerne treatments. The increase in CH_4_ emissions with the addition of *Desmanthus* in the diet might also be due to the higher quality of lucerne and to the possible differences between GEM units. The hypothesis that increasing the proportion of *Desmanthus* in the diet will reduce CH_4_ emissions when measured by GEM is rejected. Therefore, on similar high-quality diets, *Desmanthus* does not reduce CH_4_ emissions. However, *Desmanthus* can compete with a good quality legume such as lucerne in terms of DM and CP intakes. These findings could contribute to increased intakes in the drier part of northern Australia where temperate legumes such as lucerne cannot persist. Further in vivo investigation is needed to better evaluate the outdoor methane emissions in northern Australian beef cattle supplemented with *Desmanthus*.

## Figures and Tables

**Table 1:** Nutritive value (mean ± s.e.) of diets containing lucerne and *Desmanthus* spp. cultivars determined by near-infrared spectroscopy taken from Suybeng et al. [26].

Variable	Lucerne	JCU2	JCU4	JCU7	*Desmanthus* spp. ^2^	SEM	*p*-Value
CP (% DM)	10.2	9.2	10.1	9.4	9.6	0.17	0.08
ADF (% DM)	41.3 ^a^	41.9 ^b^	41.9 ^b^	42.8 ^b^	42.6	0.62	0.01
NDF (% DM)	68.4	68.7	67.2	68.8	68.3	0.74	0.13
ME (MJ/kg DM) ^1^	7.6 ^a^	7.1 ^b^	7.1 ^b^	7.0 ^b^	7.0	0.06	0.01

^1^ Estimated as DMD × 0.172 − 1.707 [31], ^2^ average of the three *Desmanthus* spp. DM = dry matter, CP = crude protein, ADF = acid detergent fiber, NDF = neutral detergent fiber, ME = metabolizable energy, SEM = standard error of the mean. Means within the same row without the same alphabetical characters (a, b) represent statistical differences (*p* < 0.05).

**Table 2:** Nutrient intakes and phenolic concentrations (±SEM) of the backgrounding, lucerne and *Desmanthus* spp. diets.

Variable	Backgrounding	Lucerne	JCU2 (*D. virgatus*)	JCU4 (*D. bicornutus*)	JCU7 (*D. Leptophyllus*)	*Des.* spp. ^1^	*p*-Value		
							Backgrounding vs. *Des* spp.	Luc. vs. *Des.* spp.	*Des.* spp.
DMI (kg/day)	5.47 ± 0.10	6.57 ± 0.10	5.96 ± 0.16	5.85 ± 0.24	5.90 ± 0.21	5.90 ± 0.12	0.01	0.02	0.96
DMI/kg LW (%)	1.99 ± 0.04	2.02 ± 0.07	2.04 ± 0.06	2.03 ± 0.05	1.96 ± 0.04	2.01 ± 0.03	0.70	0.98	0.44
CP intake (kg/day)	0.509 ± 0.01	0.705 ± 0.04	0.568 ± 0.03	0.602 ± 0.02	0.572 ± 0.03	0.582 ± 0.02	0.01	0.01	0.64
ADF intake (kg/day)	2.39 ± 0.04	2.68 ± 0.05	2.51 ± 0.06	2.40 ± 0.11	2.48 ± 0.09	2.46 ± 0.05	0.30	0.02	0.66
NDF intake (kg/day)	4.06 ± 0.08	4.37 ± 0.07	4.05 ± 0.10	3.90 ± 0.16	4.02 ± 0.13	3.99 ± 0.08	0.25	0.01	0.68
ME intake (MJ)	38.3 ± 1.24	52.1 ± 1.23	42.5 ± 1.40	43.1 ± 1.79	42.6 ± 1.62	42.8 ± 0.91	0.01	0.01	0.92
Total phenolics in diet (% DM as catechin equivalent)	0.191	0.313 ± 0.01	1.23 ± 0.10	1.42 ± 0.12	1.50 ± 0.13	1.38 ± 0.07	0.01	0.01	0.21
Condensed tannins in diet (% DM)	0.0799	0.685 ± 0.01	1.44 ± 0.13	1.64 ± 0.21	1.37 ± 0.04	1.49 ± 0.87	0.01	0.01	0.08

^1^ Average of the three *Desmanthus spp. Des. spp.* = *Desmanthus* species, Luc. = lucerne, DMI = dry matter intake, LW = liveweight, CP = crude protein, ADF = acid detergent fibre, NDF = neutral detergent fibre, ME = metabolizable energy; (±SEM) = standard error of the mean.

**Table 3:** Effect of *Desmanthus* spp. on methane emissions.

Variable	Backgrounding	Lucerne	JCU2 (*D. virgatus*)	JCU4 (*D. bicornutus*)	JCU7 (*D. leptophyllus*)	*Des.* spp. ^1^	SEM	*p*-Value		
								Backgrounding vs. *Des* spp.	Luc. vs. *Des.* spp.	*Des.* spp.
CH_4_ production (g/day)	117	137	122	121	123	122	1.93	0.01	0.01	0.92
CH_4_ yield (g/kg DMI)	21.6	21.1	21.0	20.8	21.1	21.0	0.21	0.93	0.36	0.93
CH_4_ (g/kg LW)	0.428	0.448	0.415	0.414	0.411	0.414	0.01	0.24	0.01	0.83

^1^ Average of the three *Desmanthus* spp. DMI = dry matter intake, LW = liveweight, *Des.* = *Desmanthus*, SEM = standard error of the mean.

**Table 4:** Effect of polyethylene glycol on intakes and methane emissions.

Variable	*Desmanthus* spp.		SEM	*p*-Value
	No PEG	PEG		
DMI (kg/day)	5.6	6.2	0.21	0.20
DMI/kg LW (%)	1.8	1.9	0.04	0.37
CP intake (kg/day)	0.62	0.67	0.04	0.51
ADF intake (kg/day)	2.6	2.6	0.12	0.88
NDF intake (kg/day)	4.3	4.3	0.17	0.78
ME intake (MJ)	44.8	43.6	2.19	0.91
Total phenolics in diet (%DM as catechin equivalent)	1.1	1.1	0.08	0.86
Condensed tannins in diet (% DM)	1.4	1.4	0.12	0.86
CH_4_ production (g/day)	125	145	4.16	0.03
CH_4_ yield (g/kg DMI)	22.7	23.4	0.56	0.80
CH_4_ (g/kg LW)	0.41	0.44	0.01	0.32

PEG = polyethylene glycol, DMI = dry matter intake, CP = crude protein, ADF = acid detergent fiber, NDF = neutral detergent fiber, ME = metabolizable energy, SEM = standard error of the mean, LW = liveweight.

**Table 5:** Effect of level of inclusion of *Desmanthus* cultivars on intake, methane production and yield.

	*Desmanthus* in the Diet				SEM	*p*-Value
	0	15	30	45		
DMI (kg/day)	9.4	8.9	9.0	8.6	2.34	0.16
DMI/kg LW (%)	2.26	2.19	2.23	2.11	0.02	0.77
CP intake (kg/day)	1.05	0.987	0.952	0.927	0.01	0.61
ADF intake (kg/day)	3.51 ^a^	3.33 ^ab^	3.17 ^ab^	3.01 ^b^	0.03	0.01
NDF intake (kg/day)	5.78 ^a^	5.45 ^ab^	5.13 ^b^	4.87 ^b^	0.06	0.01
ME intake (MJ)	72.7 ^a^	69.3 ^ab^	65.0 ^bc^	62.1 ^c^	0.64	0.01
CH_4_ production (g/day)	188 ^a^	232 ^b^	219 ^b^	215 ^ab^	2.88	0.01
CH_4_ yield (g/kg DMI)	20.6 ^a^	26.2 ^b^	24.2 ^c^	25.1 ^bc^	0.33	0.01
CH_4_ (g/kg LW)	0.450 ^a^	0.570 ^b^	0.545 ^ab^	0.528 ^ab^	0.01	0.01

DMI = dry matter intake, CP = crude protein, ADF = acid detergent fiber, NDF = neutral detergent fiber, ME = metabolizable energy, SEM = standard error of the mean, LW = liveweight. Means in rows with different superscripts significantly differ (*p* < 0.05).

**Table 6:** Comparison of the chemical composition and methane emissions of JCU2 and JCU4 between the three studies conducted by Durmic et al. [6], Vandermeulen et al. [5] and the current study.

	JCU2 (*D. virgatus*)			JCU4 (*D. bicornutus*)		
	Durmic et al. [6]	Vandermeulen et al. [5]	Current Study	Durmic et al. [6]	Vandermeulen et al. [5]	Current Study
CP (% DM)	16.0	12.3	10.3	17.5	16.5	13.0
NDF (% DM)	50.0	52.2	57.5	48.1	42.6	53.1
ADF (% DM)	27.2	31.2	44.5	27.1	20.5	40.4
ME (MJ/kg DM)	8.68	-	6.5	8.56	-	7.2
TP	-	4.25% DM as tannic acid equivalent	3.32% DM as catechin equivalent	-	6.90% DM as tannic acid equivalent	4.14% DM as catechin equivalent
CT	-	2.09% DM as leucocyanidin equivalent	4.10% DM	-	3.81% DM as leucocyanidin equivalent	7.27% DM
CH_4_	29.2 mL/g DMI	23.1 mL/g OM	21.0 g/kg DMI	29.7 mL/g DMI	21.9 mL/g OM	20.8 g/kg DMI

DM = dry matter, DMI = dry matter intake, CP = crude protein, ADF = acid detergent fiber, NDF = neutral detergent fiber, ME = metabolizable energy, TP = total phenolics, CT = condensed tannins, OM = organic matter.

## Data Availability

Data available on request.
